# Clinical Pharmacology of Bacteriophage Therapy: A Focus on Multidrug-Resistant *Pseudomonas aeruginosa* Infections

**DOI:** 10.3390/antibiotics10050556

**Published:** 2021-05-11

**Authors:** Dana Holger, Razieh Kebriaei, Taylor Morrisette, Katherine Lev, Jose Alexander, Michael Rybak

**Affiliations:** 1 Anti-Infective Research Laboratory, Department of Pharmacy Practice, Eugene Applebaum College of Pharmacy and Health Sciences, Wayne State University, Detroit, MI 48201, USA; dholger@wayne.edu (D.H.); R.Kebriae@wayne.edu (R.K.); Gx6737@wayne.edu (T.M.); katlev@wayne.edu (K.L.); 2 Department of Microbiology, Virology and Immunology, AdventHealth Central Florida, Orlando, FL 32803, USA; Jose.Alexander@AdventHealth.com; 3 Division of Infectious Diseases, Department of Medicine, School of Medicine, Wayne State University, Detroit, MI 48201, USA; 4 Detroit Medical Center, Department of Pharmacy, Detroit, MI 48201, USA

**Keywords:** bacteriophages, multidrug resistance (MDR), *Pseudomonas aeruginosa*

## Abstract

*Pseudomonas aeruginosa* is one of the most common causes of healthcare-associated diseases and is among the top three priority pathogens listed by the World Health Organization (WHO). This Gram-negative pathogen is especially difficult to eradicate because it displays high intrinsic and acquired resistance to many antibiotics. In addition, growing concerns regarding the scarcity of antibiotics against multidrug-resistant (MDR) and extensively drug-resistant (XDR) *P. aeruginosa* infections necessitate alternative therapies. Bacteriophages, or phages, are viruses that target and infect bacterial cells, and they represent a promising candidate for combatting MDR infections. The aim of this review was to highlight the clinical pharmacology considerations of phage therapy, such as pharmacokinetics, formulation, and dosing, while addressing several challenges associated with phage therapeutics for MDR *P. aeruginosa* infections. Further studies assessing phage pharmacokinetics and pharmacodynamics will help to guide interested clinicians and phage researchers towards greater success with phage therapy for MDR *P. aeruginosa* infections.

## 1. Introduction

For the last decade, multidrug resistant (MDR) *Pseudomonas aeruginosa* has been considered a serious threat, as defined by the Centers for Disease Control and Prevention (CDC), with an estimated 32,600 cases and 2700 associated deaths in 2017 [1]. This Gram-negative bacterial species harbors intrinsic resistance to multiple antibiotics and has developed acquired resistance to many others [2]. Similar to other Gram-negative organisms, *P. aeruginosa* exists in both planktonic and biofilm states, and it is more prone to biofilm production. Biofilms are extracellular polymeric substance matrices that embed and protect bacteria against antimicrobials and the immune system in infections such as chronically infected wounds, cystic fibrosis lung infections, and prosthetic joint infections [3]. The pathogenicity potential in combination with both intrinsic and acquired resistance mechanisms contribute to a truly arduous battle against this MDR organism. Traditionally, various antibiotics, such as beta-lactam/beta-lactamase inhibitor combinations, carbapenems, fluoroquinolones, and/or aminoglycosides, have been historically employed as the agents of choice against resistant *P. aeruginosa* isolates causing infections, but their reputation as reliable agents has been tarnished with the emergence of MDR, extensively drug-resistant (XDR) organisms, and pandrug-resistant (PDR) organisms [4]. To further complicate this issue, the antimicrobial agent pipeline for Gram-negative infections was recently described as “bleak” and “insufficient” in an analysis conducted by the World Health Organization (WHO) in 2019 [5]. Together, the lack of effective strategies to combat resistant *P. aeruginosa* organisms and poor prospects for new agents has created a great need for the development of alternative antimicrobial therapies with novel mechanisms of action, including non-antibiotic agents.

Bacteriophages (phages) are viruses that infect and replicate within bacterial cells, which in the case of lytic phages, leads to host cell death. Phages have been used to treat bacterial infections for over 100 years; however, phage therapy has largely been surpassed by antibiotics, partly due to the introduction of antibiotics, the limited activity of specific phage strains, and unfamiliarity with phage therapeutics [6,7]. Recently, in the era of antimicrobial resistance, phages have regained interest as a potential therapeutic option due to their ability to evade traditional antibiotic resistance mechanisms, the avoidance of harm to normal flora due to their specificity, and biofilm degradation mechanisms [8]. Phage therapy is primarily practiced today in parts of Eastern and Western Europe, with a research facility dedicated to phage therapeutics, the Eliava Institute, located in Tbilisi, Georgia [9]. The success of phage therapy for the treatment of *P. aeruginosa* infections in animals and humans has been validated in several case reports, as well as in small clinical and preclinical trials [10,11,12,13,14,15,16,17]. However, there remains much to be discovered in terms of the pharmacokinetics and pharmacodynamics (PK/PD) of phage therapy, specifically in the context of clinical applications and their role as combination therapy with antibiotics. Further challenges that may impede the clinical application of phage therapy, such as human response to phage therapy and the possible development of phage resistance, lie ahead [18,19].

Due to the large burden of the increasing prevalence of resistant infections, rising healthcare costs, high morbidity and mortality, and their negative impact on antimicrobial resistance and stewardship, the purpose of this review was to investigate the evidence of success and remaining challenges associated with phage therapy for MDR *P. aeruginosa* infections in order to elucidate the key pharmacological considerations of this therapeutic strategy.

## 2. Overview of *Pseudomonas aeruginosa*: Clinical Impact

The WHO dedicates their entire priority one: critical pathogen list to MDR (nonsusceptibility to ≥1 agent in ≥3 antibiotic categories) Gram-negative bacteria [20,21]. *P. aeruginosa* is a Gram-negative, non-lactose fermenting, oxidase-positive bacilli that can cause a variety of infections, such as pneumonia, urinary tract infections, surgical site infections, and bacteremia—predominately acquired within the healthcare setting [22,23]. *P. aeruginosa* is an opportunistic pathogen that is ubiquitously distributed in the environment and inhabits both soil and water [24]. In fact, hospital-acquired infections may be more frequent due to increased exposure within hospitals, where drains and sinks serve as natural reservoirs for *P. aeruginosa* [25]. Though some infections caused by relatively susceptible *P. aeruginosa* phenotypes can be treated quite straightforwardly, this pathogen has a unique capability of developing antimicrobial resistance to commonly used broad-spectrum antibiotics (such as beta-lactams, aminoglycosides, and fluoroquinolones) through numerous mechanisms that can be concurrently expressed [26].

According to the CDC’s 2019 Antibiotic Resistance Threats Report, nearly 33,000 hospitalized patients in the United States had MDR *P. aeruginosa* infections in 2017, contributing to an estimated 2700 deaths and nearly 800 million dollars in estimated attributable healthcare costs [1,27]. In numerous geographical areas, there has been an increase of MDR and XDR (XDR; nonsusceptibility to ≥ 1 agent in all but ≤2 antibiotic categories) *P. aeruginosa*, with rates of up to ~30% and ~15%, respectively [21,28,29,30]. The clinical significance of this increased prevalence of MDR/XDR *P. aeruginosa* is evident, which, coupled with the already high rate of intrinsic resistance to many commonly used antibiotics, has led to many challenging clinical scenarios. Though beta-lactamases are arguably the most clinically relevant resistance mechanism amongst MDR Gram-negative pathogens, the more common resistance mechanisms displayed by *P. aeruginosa* involve porin mutations (e.g., loss of OprD) and the upregulation of efflux pumps (e.g., MexAB-OprM) (although beta-lactamases may be expressed and overexpressed, particularly AmpC) [23,26]. This poses an obvious problem, as most of the recent novel antimicrobials developed to combat MDR Gram-negative bacteria are either primarily targeted to overcome beta-lactamases (e.g., ceftazidime/avibactam and meropenem/vaborbactam) or lack in vitro activity against *P. aeruginosa* (e.g., tigecycline and eravacycline). Furthermore, *P. aeruginosa* has been shown to exhibit phenotypic mechanisms of resistance, such as the production of biofilm, which can further compromise clinical outcomes [31].

Despite recent improvements in antibiotic development, the increasing prevalence of MDR/XDR *P. aeruginosa* complicates the choice of antibiotic regimens that optimize PK/PD properties to maximize efficacy while minimizing toxicity. More specifically, a lack of prospective trials evaluating where these agents are arguably needed the most (resistant pathogens in disease states other than urinary tract or intra-abdominal infections), a shortage of real-world observational studies, and the pipeline being at a current standstill urges the discovery of non-antibiotic options with novel mechanisms of action to combat this age of resistance that we are now facing.

## 3. *Pseudomonas* Bacteriophages Background

**Figure 1:**
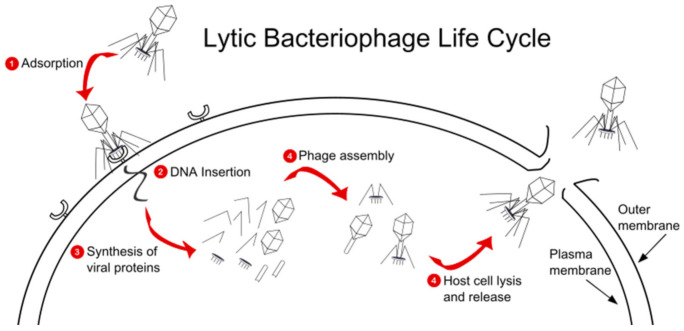
Representation of the lytic *P. aeruginosa* phage life cycle.

A possible distinct challenge with Gram-negative (in comparison to Gram-positive) phage binding may be due to the barrier of the additional outer membrane that Gram-negative pathogens express [32]. Obligately lytic (also known as virulent) *P. aeruginosa* phages are viruses that target, infect, and kill bacterial cells by taking over the bacterial machinery and, following replication, release their phage progeny to re-initiate the cycle in neighboring bacteria (Figure 1). The specific depolymerizing enzymes in lytic phages damage bacterial biofilms in *P. aeruginosa*, leading to the efficient killing of the host bacteria [33]. This is in contrast with the lysogenic (also known as temperate) lifecycle, in which the phages do not immediately kill, as they lie dormant within the bacterial cell. Given the obvious differences in activity, lytic phages are preferred to be used in the clinical setting for *P. aeruginosa* infection. Due to the intrinsic resistance to many antimicrobials that *P. aeruginosa* displays and the high rate of acquired resistance mechanisms, the study of *P. aeruginosa* phages both in vitro and in vivo is important for optimizing patient outcomes [34].

Phage therapy was first implemented in the early 1900s following its independent discovery by Frederick Twort and Felix d’Hérelle [35,36]. Though the discovery of antibiotics initially forced phage therapeutics to be neglected in the Western world, phage therapy has continued to thrive, primarily in eastern Europe. The use of phages against infections caused by *P. aeruginosa* ranges from first description in the middle of the 20th century to current recruitment in clinical trials [37,38,39]. As of 2015, ~85% of sequenced phages that target the *Pseudomonas* genus are tailed phages and are specific to *P. aeruginosa*, with the majority (~60%) being lytic. Furthermore, the majority of these phages are characterized as having long contracting tails (41%) or short non-contracting tails (38%) [40].

## 4. Benefits of *Pseudomonas* Phage Therapy

An important advantage of *P. aeruginosa* phage therapy is the ability to evade traditional mechanisms of antimicrobial resistance, as commonly seen in MDR/XDR/PDR *Pseudomonas* infections. For example, one study demonstrated the efficacy of phage ØA392 against imipenem-resistant *P. aeruginosa* bacteremia in an experimental mouse model [10], where the single-dose, intraperitoneal administration of phage ØA392 was sufficient to rescue 100% of the animals. Inherited resistance is not the only reason antibiotic therapy would fail. As mentioned previously, *P. aeruginosa* can employ a natural defense against antibiotics through the production of biofilm. Bacteria in biofilms are more refractory to antibiotics compared to those found in the planktonic state [41,42,43]. Phages, however, can produce enzymes to dissolve the biofilm matrix, an effect which has been documented in vitro using mucoid *P. aeruginosa* strains isolated from cystic fibrosis sputum samples [44,45]. In vivo success with phage therapy against biofilm-producing MDR *P. aeruginosa* was demonstrated in a case report of a patient with cystic fibrosis. Prior to phage administration, the patient had experienced persistent respiratory failure and colistin-induced renal failure as a result of previous antibiotic treatment [46]. After eight weeks of the intravenous administration of phage cocktail (AB-PA01), the patient experienced clinical resolution without the recurrence of CF exacerbation within 100 days following the end of phage therapy and successfully received lung transplantation nine months later. Furthermore, the patient experienced no adverse events associated with phage therapy, which can be partly attributed to the high specificity of phage for its target bacterial species while leaving the host microbiota unaffected [47]. Furthermore, since phages are essentially composed of proteins and nucleic acid, they are inherently non-toxic [48]—a quality providing substantial benefit over last line antimicrobial therapy, which frequently consists of nephrotoxic, colistin-based regimens, for the treatment of carbapenem-resistant, Gram-negative infections [49]. With the absence of cross-resistance to antibiotics, the ability to dissolve biofilm, and minimal potential to cause side effects, phages could be considered another potential treatment option against MDR bacteria, specifically *P. aeruginosa*.

Another challenge associated with the conventional treatment of MDR *P. aeruginosa* infections is the propensity for developing resistance to antibiotics, resulting in the emergence of XDR and PDR organisms [50,51]. As resistance to traditional antibiotics becomes more prevalent, fewer susceptible agents are left as therapeutic options. However, in anticipation of resistance development to the therapeutic agent, some researchers have targeted evolutionary trade-offs, which has often resulted in the reduced performance of another trait [52]. This strategy was successfully demonstrated in a study with several MDR *P. aeruginosa* strains, whereby the evolution of bacterial resistance to phage attack induced changes in the efflux pump mechanism, causing increased susceptibility to several antibiotic classes [52]. The study authors concluded that the *P. aeruginosa* phage OMKO1 selects against the expression of OprM and, consequently, the function of the mexAB/XY-OprM efflux systems, resulting in a significantly improved sensitivity to four drugs, including ciprofloxacin and ceftazidime. Phage–antibiotic combinations (PACs) may help to reduce the incidence of antimicrobial-resistant infections and could extend the lifetime of our current antibiotics for MDR *P. aeruginosa* infections.

## 5. Phage-Dosing Strategies for MDR *Pseudomonas aeruginosa* Infections

When administering phages for clinical application in MDR *P. aeruginosa* infections, there has yet to be formal guidance established for dosage, route of administration, and dosing frequency. Considering that phage therapy may be utilized in the form of a phage combined with antimicrobial therapy, phage dosing may ultimately depend on the indication, phage(s) host range, and specific antibiotic combinations. Multiple studies have reported success with the use of phage cocktails for MDR *P. aeruginosa* chronic wound infections; however, the dosing range greatly varies between studies for this indication, from 1 × 10^6^ PFU/mL to 1 × 10^9^ PFU/mL topically applied [14,53]. However, when applied at lower doses, such as one randomized controlled trial that topically administered 1 × 10^2^ PFU/mL to *P. aeruginosa* burn wounds every day for seven days, a longer time to sustained reduction in bacterial burden was reported in the phage-treated group in comparison to the standard of care group [54]. These examples suggest a phage-dose-related efficacy with the topical administration of phages for MDR *P. aeruginosa* wound infections, but phage-doses for other indications and routes of administration may differ.

The phage-dosing range will likely be established through reports of adverse reactions as a result of high doses of phage. One such example was recently reported in a case series from the phage institute in San Diego, California, where a patient with an MDR *P. aeruginosa* ventricular assist device (VAD) infection developed fever and wheezing related to high concentration (1 × 10^11^ PFU/mL) phage administration [12]. However, the patient experienced no such reaction after the subsequent administration of the diluted preparation (1 × 10^10^ PFU/mL). Since endotoxin concentrations in the phage preparation were well below the FDA’s allowable limit, the authors concluded there may have been additional pyrogens that were diluted upon subsequent lower concentrations of phage administration [12]. It appears that certain thresholds may exist, across which the risk of hypersensitivity reaction substantially increases, but the boundaries for safe administration remain to be defined for the treatment MDR *P. aeruginosa* infections. While the previous case utilized phage cocktails alone, several cases describe successful phage therapy in combination with antibiotics. Literature further describing the use of PAC has helped to promote this strategy as a potential option to treat MDR Gram-negative pathogens, including *Pseudomonas* [55,56,57]. All therapeutic designs (monophage, polyphage, and PAC) have reports of clinical success against *P. aeruginosa* in human applications, but the data regarding which phage-dosing strategy is preferred for MDR *P. aeruginosa* infections are still inconclusive [14,15,16,17]. While the mechanisms behind phage–antibiotic synergy (PAS) need further investigation, the synergistic effects of phages have been reported numerous times [58,59,60]. PAC therapy was found to further improve the eradication of *P. aeruginosa* infections compared to phage therapy alone in a cystic fibrosis zebrafish model [61]. An in silico simulation of phage–antibiotic combination therapy against two strains of *P. aeruginosa* demonstrated that combination therapy is superior to each agent alone for both antibiotic-resistant and phage-resistant strains, even at sub-inhibitory concentrations of antibiotics [62]. Of note, the impact of phage in PAS is not restricted to the eradication of the infection and can lead to evolutionary tradeoffs where MDR strains of *P. aeruginosa* gain more susceptibility to antibiotics [52,63]. Future phage-dosing strategies for MDR *P. aeruginosa* infections will likely include a combination of phage and antibiotics in order to attain improved eradication rates and to capitalize on evolutionary tradeoffs, which can improve susceptibility of many overutilized antibiotics.

## 6. Phage Selection for MDR *Pseudomonas aeruginosa* Infections

The abundant availability of bacteriophages in nature, both in terms of the quantity and diversity of phages, provides a large pool of options, although this may become a disadvantage given the complexity and challenges associated with phage selection [64]. In order to narrow down possible phage reservoirs when selecting phages to target MDR *P. aeruginosa*, clinically useful phages have been sourced from sampling sewage water adjacent to hospitals, as the phages found in abundance among human pathogenic bacteria are likely to use those target pathogens as hosts. As expected, phages successfully selected and isolated from sewage were shown in vitro to have a high efficacy against a variety of clinical and general laboratory strains of *P. aeruginosa* [65]. Sampled phages can be directly identified via the double drop agar method, assuming the titer is high enough or amplified for ease of identification [66]. An obvious limitation of this sourcing method is that only the phages that infect the hosts used for selection are identified, but this can allow for the categorization of a panel of newly sourced phages by their efficacy in a target pathogen, much like antibiotic susceptibility testing [67,68].

**Figure 2:**
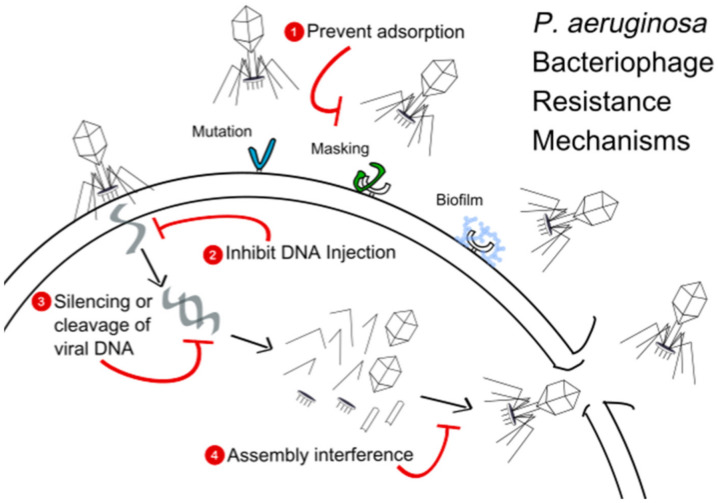
Representation of multiple *P. aeruginosa* phage resistance mechanisms.

Though phages are lauded as an alternative to target MDR *P. aeruginosa* infections, single-phage therapy also poses the risk of inducing phage resistance, a concern that may be alleviated with combining phages into a cocktail [69]. Given the multitude of mechanisms by which host organisms, such as *P. aeruginosa*, develop phage resistance (Figure 2), including the prevention of phage adsorption, the prevention of phage DNA entry, and the cutting of phage nucleic acids, it may be important to select phages for cocktails based on differing mechanisms of action to increase the likelihood of developing a counterattack to phage resistance methods [70].

Furthermore, cocktails allow for the opportunity to include phages that may otherwise be unsuitable for clinical use as a monophage therapy but that may drive the evolution of the targeted bacteria towards reduced virulence and restored antibiotic susceptibility, or they may select for a different mechanism of resistance that might allow for the more successful eradication of the infection [52,71,72,73,74]. For example, one study examined the impact of bacteriophage-resistant strains of *Pseudomonas* on bacterial virulence in ayu fish. The authors reported a reduced bacterial virulence in bacteriophage-resistant strains of *Pseudomonas* (LD_50_ > 10^4^ CFU fish-1) compared to parental strains (LD_50_ > 10^1.2^ CFU fish-1) [75]. Phage cocktails also introduce the possibility of a wider host spectrum, with the addition of phages offering a variety of host-range specificities in addition to improved killing efficiency compared to a single phage in some instances as [76,77,78]. However, one of the benefits of phage use is minimal disruption to native microflora, so there is an upper limit to the desired host range to avoid damaging non-target bacteria. That said, this impact will likely still be lower than that of typical commercial antibiotics [79]. Therefore, clinically relevant phage selection considerations should include the analysis of phages as potential candidates for use in a cocktails, as well as their efficacy when used alone against a target organism. For example, when selecting for phages in a cocktail, Lehman and colleagues considered phage host range, the frequency of resistance, and complementation [80]. Furthermore, the preclinical characterization of therapeutic phage cocktails requires answers to fundamental questions regarding their effectiveness, stability, and safety. Genomic sequencing should be performed to rule out presence of toxins, antibiotic resistance genes, etc. Lastly, their morphology and life cycle variables, such as latency time and burst size, in addition to bactericidal activity and host range, should be assessed [52,81]. Additionally, personalized, monophage therapy has been selected for the treatment of an aortic graft infected with *P. aeruginosa* based on successful in vitro experiments against a patient’s specific clinical isolate. Their results showed that antibiotics alone were not capable of reducing cell densities in biofilms, while phage OMKO1 alone or in combination with antibiotics significantly reduced bacterial densities [63]. Furthermore, in the treatment of *P. aeruginosa* biofilm-mediated infections, phage selection can be optimized to include phages that specifically target biofilm formation. Examples include phages that produce glycoside hydrolases that can target and degrade the *P. aeruginosa* exopolysaccharide biofilm matrix [82].

## 7. Pharmacokinetics of *Pseudomonas* Phage Therapy

Despite the facts that MDR *P. aeruginosa* is considered as one of the top priority pathogens and phage therapy is a promising alternative to treating these infections (due to enhanced diffusion inside biofilm matrix, site-specific action, and propagation at the infection site), data regarding the PK/PD of phage therapy are sparse, limiting the clinical applications of phage therapy [20,83,84]. Here, we have stated examples of antipseudomonal phages and their reported PK/PD in the context of general phage PK/PD, as some studies have revealed the overall principles of phage clearance regardless of the sensitive organism. The PK/PD evaluation of antipseudomonal phage ΦPEV20 (in vivo using intravenous administration in rats) showed the preferential accumulation of phage in the liver and spleen, confirming a non-homogeneous phage distribution [83]. Naturally, the spleen is the organ where active phages (both *P. aeruginosa* and non-*P. aeruginosa* phages, such as antistaphylococcal phages) can be detected for the longest time after administration. Spleen, liver, and lymph nodes have been reported as the highest phage delivery organs, while bones, joints, eyes, pancreas, inner ears, and gallbladder have shown no confirmed phage delivery when intravenously administered [85,86,87]. Consequently, spleen and liver are the major organs that filter out circulating phages. Despite the fact that renal clearance plays an important role in the removal of majority of the drugs, it was shown that there is high variability of active phage in urine of the individuals and urine titers were several orders of magnitude lower than blood titers [88]. It has also been shown that penetration to the bladder by Gram-negative phages, such as the T2 phage, is dose-dependent, and a minimum of 10^9^ PFU/mL is needed for phage detection in the urine of mice after IV administration [89]. In addition, phage concentration in plasma was dependent on both dose and the route of administration, as explained previously [90,91].

Another study on mice compromised by a burn wound injury (with fatal *P. aeruginosa* infection) showed that the route of administration was of great importance to the efficacy of treatment. Among the three tested routes of administration (intramuscular, subcutaneous, and intraperitoneal), the intraperitoneal route provided the highest protection. A significant decrease in the number of *P. aeruginosa* colonies indicated that a single dose of phage cocktail could stop the infection process before reaching the state of bacteremia and septic shock [92]. The interaction between a cocktail of three virulent phages and *P. aeruginosa* biofilms was monitored using confocal scanning microscopy. The role of parameters such as biofilm age, repeated phage treatments, and combination with sub-MIC levels of ciprofloxacin was investigated. It was reported that a combination of *P. aeruginosa* phages with antibiotics at sub-MIC levels caused a ~6-fold reduction in biofilms [93].

Similar to antipseudomonal phages, antistaphylococcal phage concentrations in murine plasma have been shown to be dose-dependent regardless of the absence or presence of sensitive bacteria [94]. Though phages are expected to be diluted and available in the body after administration, researchers have found that different routes of administration can lead to variable penetration in the blood (98.5% injection, 66.7% inhalation, 50% topical, and 41.1% oral) [95] due to phage filtration or potential phagocytosis. Gram-negative phage phagocytosis (*Escherichia coli* T2 phage) and the disintegration of phage virions [18,19,96] have been found to cause immediate reductions of phage counts in the blood after injection, as has been observed in animal models (averaging about 100-fold) [11,89,96,97,98,99,100].

Phage PK in the presence of sensitive bacteria, regardless of the susceptible organism type and genus, is fundamentally different from that of antibiotics due to phage replication at the site of infection. There are no coherent data available regarding the clearance of phage in the absence of sensitive bacteria, but many studies have considered the non-replicating phage as a conventional drug and found that the rate of non-replicating phage clearance slows down with time, suggesting that phage half-life is dose-dependent [101,102]. While there are no systematic data available on specific *P. aeruginosa* phage PK, parameters such as the extent of phage-sensitive infection, the morphology of the phage, phage phagocytosis or filtration, and phage propagation on gut bacteria can add to the complex nature of *P. aeruginosa* phage PK [103,104,105]. Gut physiology including pH (due to acidic sensitivity of the phage), coexisting microbiomes, bile inactivation, and ionic composition of the gut are critical parameters, defining phage propagation in gastrointestinal regions [95]. However, *Pseudomonas* phage KPP10, among other Gram-negative phages, has been found to be either resistant to primary bile acids or only moderately affected by incubation with bile salts [106,107].

It is of note that the immune system plays a major role in phage clearance, with phagocytosis being the main process in phage neutralization. The innate or nonspecific immune response removes the invading external elements including phages with no recognition [108,109]. An adaptive or specific immune response is an additional mechanism for phage opsonization based on phage antigens and is variable with exposure time and in different types of phages [110]. Regarding phage cocktails, antibodies induced by one phage can potentially impact the PK of another phage and the general opsonization process [95,111]. However, the impact of antibody production on phage neutralization and therapeutic effectiveness will likely depend on the timing of antibody production. For example, the intraperitoneal injection of *Pseudomonas* phages against experimental infection in mice induced phage-specific antibodies many days after the time-frame of effective treatment [95]. One potential alternative for preserving *P. aeruginosa* phages from the immune system and chemical stress while having a constant release profile is the encapsulation of phages, which allows for longer circulation in animal or human body [112,113,114].

## 8. Formulation Considerations

Another challenge associated with phage therapeutics in *P. aeruginosa* infections is the specific formulation and delivery of phage to the site(s) of infection. For example, to date, there is no consensus as to whether nebulized phage therapy is preferred over intravenous phage therapy for cystic fibrosis lung infections caused by *P. aeruginosa*. As with any therapeutic strategy, pharmacokinetic parameters and bioavailability (fraction of administered medication entering the systemic circulation) are crucial to consider in order to optimize efficacy and safety against *P. aeruginosa* infections [115]. Following standard procedures (e.g., phage isolation, screening, and purification), *P. aeruginosa* phages may be suspended in buffer or saline to be stored at appropriate temperatures or potentially processed even further by being spray-dried or encapsulated in nano- or micro-particle formulations. Other important factors to consider are the stability and shelf-life of *P. aeruginosa* phages to safeguard reproducible dosages that may be further impacted by formulation and encapsulation. For example, the nebulization of a *Pseudomonas* phage, PEV44, was shown to significantly increase the fraction of broken phages and decrease the amount of intact phages for viable phage delivery [116]. However, the encapsulation of phages in particles such as liposomes has been further shown to enhance the circulation period of phages for intraperitoneal therapy [113].

A pharmaceutical formulation often functions in a manner that includes various dosage forms and can lead to varying PK [117]. Numerous dosage formulations have been utilized in the clinical setting with phages, with the most common being oral, intravenous, and topical/local formulations; however, the scientific and clinical community have yet to determine the most optimal formulation(s) (or a combination) for *P. aeruginosa* infections and various infectious sites [118]. *P. aeruginosa* phages have been used in the clinical setting for *P. aeruginosa* infections, with most of the aforementioned formulations being utilized [12,13,63]. Wright and colleagues conducted the first randomized, double-blind, placebo-controlled phase I/II clinical trial that evaluated 24 patients with chronic otitis caused by antibiotic-resistant *P. aeruginosa*. Their utilized phage preparation consisted of a locally administered phage preparation and showed preliminary efficacy and safety in the treated patients [13]. Chan and colleagues reported the successful use of *P. aeruginosa* phage solution OMKO1 (in combination with ceftazidime) administered into the mediastinal fistula in a chronic infection of an aortic graft with associated aorto-cutaneous fistula [63]. A recent report from Aslam and colleagues reported the first ten consecutive cases of intravenous phage therapy to treat MDR bacterial infections in the United States. Overall, the most prevalent request (14.3%) and utilization (40%) were for *P. aeruginosa* phages, and those patients that received therapy included a 67-year-old male with a previous lung transplant and pneumonia (nebulized and IV phage), a 26-year-old female with cystic fibrosis who developed pneumonia (IV phage), 60-year-old and 82-year-old males with a ventricular assist device infection (both IV phage), and a 64-year-old male with recurrent bacteremia and probable aortic graft infection (IV phage) [12].

It has been noted that a number of phase I and phase II trials of phage therapy have shown preliminary efficacy without notable safety concerns. Vandenheuval and colleagues reported that most clinical trials have utilized phage suspensions and have not processed phage cocktails into specific dosage formulations [119]. It is of utmost importance that differing formulations of phages are critically evaluated in the clinical setting to determine which (if any) phage formulations prove to be superior and whether this varies based on pathogen and/or infection type.

## 9. Efficacy of Phage Therapy against MDR *Pseudomonas aeruginosa*

Previously, the success of phage therapy for MDR *P. aeruginosa* infections in human application has generally been limited to salvage therapy and case reports. Even though phage therapy for *P. aeruginosa* infections has been used routinely in parts of eastern Europe, little peer-reviewed data are available from this region [120]. However, in recent years, more human evaluations with phage therapy for MDR *P. aeruginosa* infections have accumulated in the form of limited case series and randomized controlled trials (Table 1). For example, the first phase I randomized controlled trial focused on phage therapy examined the safety of topical phage application for the treatment of venous leg ulcers caused by *P. aeruginosa* and other organisms [53]. However, limitations of the trial included inability to detect efficacy due to insufficient power and a lack of requirement for in vitro susceptibility testing to the phage preparation. Another phase I/II trial was able to measure the efficacy of a phage cocktail that was topically applied for the treatment of burn wounds in 25 patients, but no significant difference in efficacy was identified when comparing phage therapy and standard of care. It is important to note that the applied phage concentration was significantly lower than dose concentrations utilized in similar investigational studies with topical phage application [15,53,121]. Due to the lack of randomized controlled trial data specific to *P. aeruginosa* phage therapy, there has been significant variability reported in terms of phage concentration doses, therapy duration, and frequency of application between studies and within study protocols (see Table 1). Most human trial data have thus far come from topical application studies; however, there are emerging new data regarding alternate routes of administration, including intravenous and oral, which may prove more effective for systemic MDR *P. aeruginosa* infections.

Reported recently from a single center in San Diego, CA, a case series of 10 patients detailed the successes and failures of phage therapy via the intravenous route against various resistant organisms, with MDR *P. aeruginosa* representing 5 out of 10 cases [12]. The center utilized the susceptibility testing of bacteria to phage for each patient isolate, but the authors noted two cases of MDR *P. aeruginosa* infection in which the treatment failure of VAD occurred despite initial susceptibility to the phage. On the other hand, three patients experienced the clinical resolution of their MDR *P. aeruginosa* infection (two cases of pneumonia and one case of recurrent bacteremia). Interestingly, the initial dose concentrations administered in the two treatment failures for VAD infections (range: 10^5^–10^7^ PFU/mL) were significantly lower than those administered in the successful *P. aeruginosa* pneumonia cases (>10^9^ PFU/mL) [12]. These findings suggested the need to optimize phage therapy regarding dose, route, and frequency of administration for each clinical indication. Likely, the efficacy associated with increased dose concentration depends on the infection being treated, as well as the presence of biofilm in the associated *P. aeruginosa* infection.

## 10. Remaining Gaps in Literature

Though some investigational studies have demonstrated the safety [12,16,46] and efficacy [54,63] of bacteriophage therapy against *P. aeruginosa* infection, many questions remain. The lack of randomized controlled trials has driven the generation of case reports and series, limiting the potential for extrapolation of findings related to phage therapy alone. Adding to this problem, the emergence of contradicting results has established the need for further investigation into phage therapeutics such as optimal dosing, PK/PD, and administration techniques specific to MDR *P. aeruginosa* infections [53]. Beginning with the *P. aeruginosa* phage mechanism of action, it is still unclear whether phage antibacterial activity may be partly attributed to their recruitment of the immune system. Both natural phages (e.g., in nature or in the gut) and exogenous (e.g., the ones introduced to patients for therapeutic reasons) are in constant interaction with mammalian immune system. Though many in vitro investigations have reported specific antibody production as a result of *P. aeruginosa* phage activity, the mechanistic insights of this process are not clear [124,125,126,127,128,129,130]. Examples of this effect include the induction of anti-inflammatory cytokine IL-10 by all tested *Pseudomonas* phages but not by any staphylococcal phage. On the other hand, the latter phage induced TNFα, whereas only two out of four *Pseudomonas* phages produced this effect [131]. There are contradicting in vivo data that have demonstrated the inhibition of TNF production and phagocytosis with a filamentous *Pseudomonas* phage, while another Gram-negative phage did not produce such effects [130,132]. Similarly, the production of phage-neutralizing antibodies, such as IgG, IgM, and IgA, were induced upon exposure to therapeutic doses of phages, but no correlation between therapeutic outcomes and the number of antibodies was observed [133,134]. One in vivo study examined phage therapy for acute pneumonia caused by MDR *P. aeruginosa* in a mouse model to further understand the impact of host immunity on efficacy [135]. Their results showed the successful clearing of acute respiratory infections with the required assistance of host innate immunity, specifically neutrophils, a relationship the authors coined “immunophage synergy.” Additionally, phage suspensions active against *P. aeruginosa*, among other organisms, have been shown to reduce inflammatory markers (C-reactive protein and sedimentation rate) in vivo, which has led to the consideration of the therapeutic potential for phage therapy in autoimmune liver diseases [136,137]. However, the role of phage anti-inflammatory properties in the treatment of MDR *P. aeruginosa* infections is not fully understood.

In addition to the full elucidation of the mechanism of *P. aeruginosa* phage therapy including the recruitment of the human immune system, questions regarding optimal dose, frequency, and route of administration remain. In order to answer these questions, the PK/PD of phage therapy should be further investigated. Though not specific to *P. aeruginosa*, there is one randomized, double-blind controlled trial in the recruitment process that aims to evaluate the PK/PD of the phage cocktail, LBP-EC01, in patients colonized with *Escherichia coli* [138]. This study will be the first randomized controlled trial to assess the PK/PD of intravenous Gram-negative phage therapy, which will help provide insight into optimal dosing strategies and answer questions relating to the administration of *Pseudomonas* phages. However, the assessment of phage PK in the presence of antibiotics remains to be investigated in randomized controlled trials. The production of outer membrane vesicles (OMVs) by *P. aeruginosa* strains may also contribute towards phage dosing. In vitro studies on various Gram-negative organisms have suggested that OMVs reduce bacterial killing by phage in a dose-dependent manner [139,140]. Perhaps *Pseudomonas* strains producing high amounts of OMVs will require a different phage-dosing scheme compared to those strains will lower OMV production rates.

As previously mentioned, phage cocktails for MDR *P. aeruginosa* have emerged as a popular option, but the selection criteria for phages within the cocktail have not been defined in a standard, repeatable process. Many in vivo trials evaluating *Pseudomonas* phage cocktails have chosen specific phages based on broad host range only. However, recent in vitro studies have suggested that selecting phages based on plaque size may be more appropriate. One in vitro study observed an enhanced biofilm inhibition of *P. aeruginosa* when selecting for phages that produce plaques with a diameter of ≤0.5 mm, as compared to those with larger plaque sizes [141]. Previous literature has described the plaque size as a phenotype representative of phage adsorption rate, that is, smaller plaque size equates to higher adsorption rate [142]. Therefore, it is possible that with quicker adsorption to a host organism, a phage is more effectively able to inhibit host organism matrix formation. It is important to note that when comparing plaque size results, all experiments should be performed in the same conditions (i.e., temperature, percentage of overlay agar, and the amount of specific ions in the agar) as any of the aforementioned parameters can manipulate plaque size [142]. More in vivo trials will be needed to further assess the relationship between phage adsorption rate and biofilm inhibition in *P. aeruginosa* infections.

## 11. Challenges Ahead

### 11.1. Possibility of the Infected Organism Acquiring Virulence Traits from the Phage

Despite the promising data highlighted in the previous sections of this review, phage therapy against *P. aeruginosa* has not been brought to mainstream medicine, especially in Western countries [143,144,145]. One of the main challenges regarding phage therapy for *P. aeruginosa* is the biofilm-forming nature of this organism, especially in conditions such as cystic fibrosis (CF) lung infection. There is a paucity of clinical in vitro models mimicking the mucoid structure of *P. aeruginosa*. Another complication in cases like CF is the fact that biofilm-forming and non-biofilm-forming microbial communities co-exist and create co-evolving microbial and phage communities that can potentially impact phage-induced responses [146,147]. From the evolutionary standpoint, continuous gene exchange and recombination between viral and host DNA drives the fitness of bacterial communities [148].

Comparative genomics have shown that the chromosomes of bacteria and their viruses (phages) are co-evolving [149]. This development is most evident in bacterial communities where the majority of the population contain prophages or phage remnants integrated into bacterial DNA [149,150,151]. In this context, temperate phages seem to play significant roles in bacterial evolution, since, as prophages, they are able to establish long-term genetic associations with their hosts [152]. Regardless of their cycle (lytic, temperate, or lysogenic), phages contribute to the pathogenicity of their bacterial hosts through the transfer of genetic material to bacterial cell via phage infection [153]. The devotion of bacterial resources to the production of virions is not advantageous to the bacterium. However, a subset of bacteriophage genomes encrypt virulence factors (VF) to the bacteria, and the production of these VFs can augment bacterial survival through enhanced bacterial fitness [154].

Of note, most mobile antibiotic resistance genes are encoded on plasmids or transposons, and no examples of phage-encoded resistance genes have been reported. However, phages may play a key role via transduction in the mobility of these resistance plasmids among bacterial populations [155]. This phenomenon began to be reported as early as 1927; however, there was an absence of mechanistic explanations for these observations, and early investigators hypothesized that bacteria acquire virulence properties over time [156]. Recently, researchers have identified phage–bacteria interactions in bacterial host cells that lead to the virulence of bacterial pathogens [154]. One of the first examples of this phenomenon was observed in *Corynebacterium diphtheriae*, where a phage encoded the diphtheria toxin to the genome of bacteria [157]. Another example is the *P. aeruginosa* ctx-encoding cytotoxin, which is carried by a temperate phage ΨCTX [158]. A critical step towards designing *Pseudomonas* phage clinical trials would be characterizing phage-specific parameters such as latency, burst size, and affinity towards various *P. aeruginosa* receptors. In addition, those studies should monitor and detect phages carrying undesirable genes coding for toxins and antibiotic resistance [76,84,159,160].

Though numerous toxin genes were found to be phage-encoded, there is strong evidence that toxin genes are not the only VFs encoded by phages to their hosts. According to previous literature, there are at least four potential mechanisms contributing to the phage-encoding of bacterial VFs [152]. Among those, enhanced gene mobility results in the majority of VF gene transfers within bacterial communities [152]. Even though exotoxin production was historically assumed to be associated with phage infection, additional VFs have been found to be associated with phages [155]. Phages are capable of altering all aspects of host bacterial pathogenesis relevant to all stages of the infectious process including bacterial adhesion, colonization, invasion and spread through human tissues, resistance to immune defenses, and sensitivity to antibiotics [155]. For instance, phage FIZ15 promotes adhesion to buccal epithelial cells [161]. In addition, when *P. aeruginosa* strain 1 is lysogenized with phage D3, somatic antigens of the bacterium are modified, which leads to a loss of opsonization by peritoneal macrophages in vitro [162]. Though lysogenic pathways may not appear to be clinically critical, they can encode certain VFs that contribute to evolutionary alterations in the bacterium genome and create additional level fitness against antibacterial agents [155]. Further research regarding administration routes should consider spray-dried respirable powders, suspensions, or nebulized phages—especially in cases such as CF [163,164].

### 11.2. Adsorption Inhibition

One of the remarkable challenges in phage therapy is adsorption inhibition. For instance, colistin causes *P. aeruginosa* cell death by destabilizing the cell membrane and thus limiting phage propagation [146]. Even though phage binding to the surface of the bacterium is considered as an energy-independent process, the consequential steps in the life of phage are energy-dependent. Previous researchers showed that although phage PL-1 was adsorbed on the surface of starved host cells, the next step of DNA injection was not achieved in the absence of an active cell metabolism (i.e., a reduction in intracellular ATP content) [165]. Typically, various components present in the phage–bacterium environment can impact phage adsorption, as phage propagation is strongly dependent on the physiological state of the host. The concept of physiological refuge refers to a phage-sensitive host that gains transient resistance to phage infection due to starvation or other environmental factors [166]. For example, immunoglobulin G has shown to have a significant inhibiting effect on the adsorption of phages to staphylococci [167]. Similarly, temperature significantly restricts the adsorption of *Listeria* phages. This is due to the activation/de-activation of host receptors at certain temperatures [166]. Starvation conditions, such as a lack of nitrogen in a broth, have been found to inhibit phage adsorption and propagation in *Lactobacillus Plantarum* ATCC 8014 [165]. Another report postulated that a common site on the cell envelope (a protein involved in iron transport) of *Salmonella typhimurium* binds to both ferrichrome and phage ES18 [168].

The evolutionary adaptation of *P. aeruginosa* bacteria to phages is another reason for reduced phage adsorption, as described previously. During this process, *P. aeruginosa* bacteria implement multiple mechanisms such as the inhibition of phage adsorption to prevent phage infection. For instance, a resistant mutant of *P. aeruginosa* strain PA1 gained resistance to phage PaP1 due to the elimination of long chain o-antigen on its cell envelope [169].

## 12. Potential of *Pseudomonas* Phage Therapy

Outside of laboratory conditions, *P. aeruginosa* rarely exists in planktonic state in a liquid environment. These bacteria exist as colonies integrated in 3D structure polysaccharide metrices called biofilms. This structure plays an important role in protecting the bacteria from antibiotic diffusion/binding, and biofilm cells are generally more refractory/resistant to antibiotics [43,170]. Phages have proven to have enhanced activity in vitro against biofilm populations of *P. aeruginosa*, thus offering great potential for use in biofilm-producing *P. aeruginosa* infections. One study demonstrated single regimens of phage or antibiotic (five different classes) only had minimal activities against biofilms; however, certain combination regimens caused synergy. Though not clinically relevant (patients receive antibiotics, if any, prior to phage treatment), *P. aeruginosa* phage treatment before antibiotic treatment achieved maximal eradication [171]. Due to fundamental differences in the mechanisms of action of phages versus antibiotics in the 3D structure of biofilm matrixes and the poor diffusion of antibiotics, phages are viable alternatives for treating biofilm infections [41,170,172]. These include prosthetic joint infections (PJI), which often result in treatment failure due to biofilm formation on implant surfaces and the adherence of biofilm bacteria on bone next to the implant [173]. PJIs are one of the most serious complications after total joint replacement (TJR), with rates as high as 14% with revision surgeries [174]. With an aging population and an increasing need for total join replacements (TJR), the number of TJR surgeries and associated PJI is expected to increase over the next few years. Success rates with traditional antibiotics yield an average treatment success rate of 33% for knee infections and 52% for hip and knee infections [174]. One case study described the use of phage therapy for a complex bone and joint infection due to XDR *P. aeruginosa*, where combination therapy with ceftolozane/tazobactam, colistin, and phages resulted in rapid wound healing and a cleared culture by day 14 [175]. Therefore, therapeutic potential may exist for phage therapy in the treatment of biofilm and prosthetic joint infections caused by MDR *P. aeruginosa*.

Similarly, chronic infection with *Pseudomonas* in patients with CF is associated with poor lung function and increased mortality [176,177,178]. Even with standard antibiotic eradication protocols, success is often variable and not sustained, which provides another area of potential for *Pseudomonas* phage therapy. As described previously, phage therapy has been successfully utilized for both in vitro and in vivo studies with successful outcomes in patients with cystic fibrosis and pneumonia [12,61,179]. One successful phage cocktail, AP-PA02, can kill more than 80% of *Pseudomonas* strains from patients with CF [179]. The safety and tolerability of an inhaled AP-PA02 cocktail formulation will be evaluated an upcoming phase 1/2 clinical trial for patients with chronic *P. aeruginosa* lung infections and CF [180]. Needless to say, there are expanding opportunities for clinical phage application in patients with cystic fibrosis and chronic infection with MDR *P. aeruginosa*.

## 13. Concluding Remarks

The first dedicated phage therapy center in North America—Innovative Phage Applications and Therapeutics (IPATH)—has received more requests for phage therapy against *P. aeruginosa* than any other single organism at their institution [12]. The need for bacteriophage therapy for MDR/XDR *P. aeruginosa* has grown during the era of antimicrobial resistance in the setting of a dry pipeline for antibiotics [12,181]. Clinical reports of bacteriophage therapy as salvage therapy have accumulated and provided researchers with a foundation of data for the development of critical questions regarding optimal dose, route of administration, and therapeutic strategy. Promising results of therapeutic successes of phage therapy for systemic *P. aeruginosa* infections will likely provide incentive for further pharmaceutical investment into human clinical trials with phage cocktails and phage–antibiotic combinations, which are greatly needed in order to implement phage therapy as a standard treatment option. With a growing body of evidence, phage therapy has the potential to serve as an effective alternative or combination agent for patients with MDR and XDR *P. aeruginosa* infections.

## Figures and Tables

**Table 1:** Summary of the most relevant studies with targeted *P. aeruginosa* phage therapy in humans.

Indication	Phage Dose	Phage	*n*	Main Outcome	Ref.
Randomized Controlled Trials					
Leg ulcers	4 mL (1 × 10^9^ PFU/mL) topically applied once weekly for 12 weeks	Phage cocktail (WPP-201)	42	Phase I safety trial: no significant difference was reported between the phage-treated group and the control group for the frequency of adverse events or the frequency of healing.	[53]
Burn wounds	1 mL (1 × 10^2^ PFU/mL), topically applied once daily for 7 days	Phage cocktail (PP1131)	27	Patients in the phage group experienced a longer time to sustained reduction in bacterial burden compared to the SOC group (144 vs. 47 h).	[54]
Chronic otitis	0.2 mL (6 × 10^4^ PFU) of antibiotic application once	Phage cocktail (Biophage-PA)	24	Phase I/II controlled clinical trial: statistically significant clinical improvements from baseline in the phage-treated group compared with the control group.	[13]
Prospective Trials					
Burn wounds	1 mL (10^9^ PFU/mL) per 50 cm^2^ topically applied once	Phage cocktail (BFC-1)	9	For all patients, the bacterial load remained unchanged after phage application, as well as after standard treatment.	[121]
Case Series					
Skin ulcers and wounds	1 × 10^6^ PFU/cm^2^ topically applied until ulcer healed (6 days to 15 months)	Phage cocktail (Pyophage)	96	Wounds/ulcers completely healed in 70% of patients.	[14]
Septicemia	10 mL (PFU/mL: NR) orally three times daily (median duration: 29 days)	NR	94	Complete recovery in 80 patients. Phage therapy was ineffective in 14 patients. No significant difference reported for phage therapy alone (*n* = 23) or PAC (*n* = 71).	[16]
Systemic infections	2 × 10^5^–4 × 10^10^ PFU/mL IV +/− nebulization for 4–12 weeks	Phage cocktail	5	Intravenous BT was safe, with a successful outcome in 3/5 patients with antibiotic-recalcitrant *P. aeruginosa* infections.	[12]
Chronic wounds	0.1 mL/cm^2^ (1 × 10^9^ PFU/mL) topically applied on alternate days x 3–5 doses	Phage cocktail	20	All wounds became sterile within 13 days, and 7 cases achieved complete wound healing by day 21.	[15]
Systemic infections	1 × 10^8^ PFU/mL orally TID for 2–9 weeks (median duration: 32 days)	Single phage or phage cocktail	20	The cure of infection was achieved in all cases.	[17]
Case Reports					
CF pneumonia	5 mL (4 × 10^9^ PFU/5 mL) IV every 6 h for 8 weeks	Phage cocktail (AB-PA01)	1	Clinical resolution of infection without the recurrence of pneumonia due to *Pseudomonas* or CF exacerbation within 100 days following the end of BT.	[46]
Septicemia and wounds	50 mL (10^9^ PFU/mL) of IV infusion once daily and 50 mL of irrigation every 8 h for 10 days	Phage cocktail (BFC-1)	1	Pathogen eradicated from blood, CRP levels dropped, fever disappeared, and kidney function returned after a few days.	[122]
Burn wound	0.2 mL (1 × 10^3^ PFU/mL) topically applied once	NR	1	Three days after phage application, *P. aeruginosa* was not isolated from tissue swabs. Extensive grafting following phage therapy was successful.	[123]
Aortic graft infection	10 mL (1 × 10^7^ PFU/mL) one injection into fistula	Phage OMKO1	1	Following the application of phage and ceftazidime, the infection appeared to resolve with no signs of recurrence.	[63]

Abbreviations: PFU: plaque forming units; PAC: phage–antibiotic combination; CRP: C-reactive protein; BT: bacteriophage therapy; SOC: standard of care; NR: not reported; TID: three times daily; IV: intravenous; CF: cystic fibrosis.

## References

[1] CDC (2019). Antibiotic Resistance Threats in the United States.

[2] Bonomo R.A., Szabo D. (2006). Mechanisms of Multidrug Resistance in Acinetobacter Species and *Pseudomonas aeruginosa*. Clin. Infect. Dis..

[3] Tkhilaishvili T., Wang L., Perka C., Trampuz A., Gonzalez Moreno M. (2020). Using Bacteriophages as a Trojan Horse to the Killing of Dual-Species Biofilm Formed by *Pseudomonas aeruginosa* and Methicillin Resistant *Staphylococcus aureus*. Front. Microbiol..

[4] Sader H.S., Castanheira M., Duncan L.R., Flamm R.K. (2018). Antimicrobial Susceptibility of Enterobacteriaceae and *Pseudomonas aeruginosa* Isolates from United States Medical Centers Stratified by Infection Type: Results from the International Network for Optimal Resistance Monitoring (INFORM) Surveillance Program, 2015–2016. Diagn. Microbiol. Infect. Dis..

[5] World Health Organization (2019). 2019 Antibacterial Agents in Clinical Development: An Analysis of the Antibacterial Clinical Development Pipeline.

[6] Hill C., Mills S., Ross R.P. (2018). Phages & antibiotic resistance: Are the most abundant entities on earth ready for a comeback?. Future Microbiol..

[7] Wittebole X., Roock S.D., Opal S.M. (2014). A historical overview of bacteriophage therapy as an alternative to antibiotics for the treatment of bacterial pathogens. Virulence.

[8] Taati Moghadam M., Khoshbayan A., Chegini Z., Farahani I., Shariati A. (2020). Bacteriophages, a New Therapeutic Solution for Inhibiting Multidrug-Resistant Bacteria Causing Wound Infection: Lesson from Animal Models and Clinical Trials. Drug Des. Dev. Ther..

[9] Myelnikov D. (2018). An Alternative Cure: The Adoption and Survival of Bacteriophage Therapy in the USSR, 1922–1955. J. Hist. Med. Allied Sci..

[10] Antoine C., Laforêt F., Blasdel B., Glonti T., Kutter E., Pirnay J.P., Mainil J., Delcenserie V., Thiry D. (2021). Efficacy assessment of PEV2 phage on Galleria mellonella larvae infected with a *Pseudomonas aeruginosa* dog otitis isolate. Res. Vet. Sci..

[11] Chen F., Cheng X., Li J., Yuan X., Huang X., Lian M., Li W., Huang T., Xie Y., Liu J. (2021). Novel Lytic Phages Protect Cells and Mice against *Pseudomonas aeruginosa* Infection. J. Virol..

[12] Aslam S., Lampley E., Wooten D., Karris M., Benson C., Strathdee S., Schooley R.T. (2020). Lessons Learned from the First 10 Consecutive Cases of Intravenous Bacteriophage Therapy to Treat Multidrug-Resistant Bacterial Infections at a Single Center in the United States. Open Forum Infect. Dis..

[13] Wright A., Hawkins C.H., Anggård E.E., Harper D.R. (2009). A controlled clinical trial of a therapeutic bacteriophage preparation in chronic otitis due to antibiotic-resistant *Pseudomonas aeruginosa*; a preliminary report of efficacy. Clin. Otolaryngol..

[14] Markoishvili K., Tsitlanadze G., Katsarava R., Glenn J., Sulakvelidze A. (2002). A novel sustained-release matrix based on biodegradable poly(ester amide)s and impregnated with bacteriophages and an antibiotic shows promise in management of infected venous stasis ulcers and other poorly healing wounds. Int. J. Dermatol..

[15] Gupta P., Singh H.S., Shukla V.K., Nath G., Bhartiya S.K. (2019). Bacteriophage Therapy of Chronic Nonhealing Wound: Clinical Study. Int. J. Low. Extrem. Wounds..

[16] Weber-Dąbrowska B., Mulczyk M., Górski A. (2003). Bacteriophages as an efficient therapy for antibiotic-resistant septicemia in man. Transplant. Proc..

[17] Weber-Dabrowska B., Mulczyk M., Górski A. (2001). Bacteriophage therapy for infections in cancer patients. Clin. Appl. Immunol. Rev..

[18] Jończyk-Matysiak E., Weber-Dąbrowska B., Owczarek B., Międzybrodzki R., Łusiak-Szelachowska M., Łodej N., Górski A. (2017). Phage-Phagocyte Interactions and Their Implications for Phage Application as Therapeutics. Viruses.

[19] Kantoch M. (1961). The role of phagocytes in virus infections. Arch. Immunol. Ther. Exp..

[20] WHO Publishes List of Bacteria for which New Antibiotics are Urgently Needed. https://www.who.int/news-room/detail/27-02-2017-who-publishes-list-of-bacteria-for-which-new-antibiotics-are-urgently-neneed.

[21] Magiorakos A.P., Srinivasan A., Carey R.B., Carmeli Y., Falagas M.E., Giske C.G., Harbarth S., Hindler J.F., Kahlmeter G., Olsson-Liljequist B. (2012). Multidrug-resistant, extensively drug-resistant and pandrug-resistant bacteria: An international expert proposal for interim standard definitions for acquired resistance. Clin. Microbiol. Infect..

[22] Daly J.A., Boshard R., Matsen J.M. (1984). Differential primary plating medium for enhancement of pigment production by *Pseudomonas aeruginosa*. J. Clin. Microbiol..

[23] Lister P.D., Wolter D.J., Hanson N.D. (2009). Antibacterial-Resistant *Pseudomonas aeruginosa*: Clinical Impact and Complex Regulation of Chromosomally Encoded Resistance Mechanisms. Clin. Microbiol. Rev..

[24] Diggle S.P., Whiteley M. (2020). Microbe Profile: *Pseudomonas aeruginosa*: Opportunistic pathogen and lab rat. Microbiology.

[25] Lalancette C., Charron D., Laferrière C., Dolcé P., Déziel E., Prévost M., Bédard E. (2017). Hospital Drains as Reservoirs of *Pseudomonas aeruginosa*: Multiple-Locus Variable-Number of Tandem Repeats Analysis Genotypes Recovered from Faucets, Sink Surfaces and Patients. Pathogens.

[26] O’Donnell J.N., Bidell M.R., Lodise T.P. (2020). Approach to the Treatment of Patients with Serious Multidrug-Resistant *Pseudomonas aeruginosa* Infections. Pharmacother. J. Hum. Pharmacol. Drug Ther..

[27] Healthcare-Associated Infections—Community Interface (HAIC): Multi-site Gram-Negative Surveillance Initiative. https://www.cdc.gov/hai/eip/mugsi.html.

[28] Peña C., Cabot G., Gómez-Zorrilla S., Zamorano L., Ocampo-Sosa A., Murillas J., Almirante B., Pomar V., Aguilar M., Granados A. (2015). Influence of Virulence Genotype and Resistance Profile in the Mortality of *Pseudomonas aeruginosa* Bloodstream Infections. Clin. Infect. Dis..

[29] Sader H.S., Huband M.D., Castanheira M., Flamm R.K. (2017). Pseudomonas aeruginosa Antimicrobial Susceptibility Results from Four Years (2012 to 2015) of the International Network for Optimal Resistance Monitoring Program in the United States. Antimicrob. Agents Chemother..

[30] Walkty A., Lagace-Wiens P., Adam H., Baxter M., Karlowsky J., Mulvey M.R., McCracken M., Zhanel G.G. (2017). Antimicrobial susceptibility of 2906 Pseudomonasaeruginosa clinical isolates obtained from patients in Canadian hospitals over a period of 8 years: Results of the Canadian Ward surveillance study (CANWARD), 2008–2015. Diagn. Microbiol. Infect. Dis..

[31] Donlan R.M. (2002). Biofilms: Microbial Life on Surfaces. Emerg. Infect. Dis..

[32] Jakutytė L., Baptista C., São-José C., Daugelavičius R., Carballido-López R., Tavares P. (2011). Bacteriophage Infection in Rod-Shaped Gram-Positive Bacteria: Evidence for a Preferential Polar Route for Phage SPP1 Entry in Bacillus subtilis. J. Bacteriol..

[33] Criscuolo E., Spadini S., Lamanna J., Ferro M., Burioni R. (2017). Bacteriophages and Their Immunological Applications against Infectious Threats. J. Immunol. Res..

[34] Jurczak-Kurek A., Gąsior T., Nejman-Faleńczyk B., Bloch S., Dydecka A., Topka G., Necel A., Jakubowska-Deredas M., Narajczyk M., Richert M. (2016). Biodiversity of bacteriophages: Morphological and biological properties of a large group of phages isolated from urban sewage. Sci. Rep..

[35] Duckworth D.H. (1976). Who discovered bacteriophage?. Bacteriol. Rev..

[36] Herelle F., Smith G.H. (1922). The Bacteriophage, Its Rôle in Immunity.

[37] Kellenberger G., Kellenberger E. (1957). Electron microscopical studies of phage multiplication: III. Observation of single cell bursts. Virology.

[38] Holloway B.W., Egan J.B., Monk M. (1960). Lysogeny in Pseudomonas Aeruginosa. Aust. J. Exp. Biol. Med. Sci..

[39] ClinicalTrials.gov. https://clinicaltrials.gov/ct2/show/NCT04803708.

[40] Pires D.P., Boas D.V., Sillankorva S., Azeredo J. (2015). Phage Therapy: A Step Forward in the Treatment of *Pseudomonas aeruginosa* Infections. J. Virol..

[41] Davies D. (2003). Understanding biofilm resistance to antibacterial agents. Nat. Rev. Drug Discov..

[42] Kirby A.E., Garner K., Levin B.R. (2012). The Relative Contributions of Physical Structure and Cell Density to the Antibiotic Susceptibility of Bacteria in Biofilms. Antimicrob. Agents Chemother..

[43] Costerton J.W., Stewart P.S., Greenberg E.P. (1999). Bacterial biofilms: A common cause of persistent infections. Science.

[44] Chang R.K., Das T., Manos J., Kutter E., Morales S., Chan H.K. (2019). Bacteriophage PEV20 and ciprofloxacin combination treatment enhances removal of P. aeruginosa biofilm isolated from cystic fibrosis and wound patients. AAPS J..

[45] Alemayehu D., Casey P.G., McAuliffe O., Guinane C.M., Martin J.G., Shanahan F., Coffey A., Ross R.P., Hill C. (2012). Bacteriophages ϕMR299-2 and ϕNH-4 Can Eliminate *Pseudomonas aeruginosa* in the Murine Lung and on Cystic Fibrosis Lung Airway Cells. mBio.

[46] Law N., Logan C., Yung G., Furr C.L., Lehman S.M., Morales S., Rosas F., Gaidamaka A., Bilinsky I., Grint P. (2019). Successful adjunctive use of bacteriophage therapy for treatment of multidrug-resistant *Pseudomonas aeruginosa* infection in a cystic fibrosis patient. Infection.

[47] Skurnik M., Pajunen M., Kiljunen S. (2007). Biotechnological challenges of phage therapy. Biotechnol. Lett..

[48] Loc-Carrillo C., Abedon S.T. (2011). Pros and cons of phage therapy. Bacteriophage.

[49] Tamma P.D., Aitken S.L., Bonomo R.A., Mathers A.J., van Duin D., Clancy C.J. (2020). Infectious Diseases Society of America Antimicrobial Resistant Treatment Guidance: Gram-Negative Bacterial Infections. Clin. Infect. Dis..

[50] Mentzelopoulos S.D., Pratikaki M., Platsouka E., Kraniotaki H., Zervakis D., Koutsoukou A., Nanas S., Paniara O., Roussos C., Giamarellos-Bourboulis E. (2007). Prolonged use of carbapenems and colistin predisposes to ventilator-associated pneumonia by pandrug-resistant *Pseudomonas aeruginosa*. Intensive Care Med..

[51] Falagas M.E., Bliziotis I.A., Kasiakou S.K., Samonis G., Athanassopoulou P., Michalopoulos A. (2005). Outcome of infections due to pandrug-resistant (PDR) Gram-negative bacteria. BMC Infect. Dis..

[52] Chan B.K., Sistrom M., Wertz J.E., Kortright K.E., Narayan D., Turner P.E. (2016). Phage selection restores antibiotic sensitivity in MDR *Pseudomonas aeruginosa*. Sci. Rep..

[53] Rhoads D., Wolcott R., Kuskowski M., Wolcott B.M., Ward L.S., Sulakvelidze A. (2009). Bacteriophage therapy of venous leg ulcers in humans: Results of a phase I safety trial. J. Wound Care..

[54] Jault P., Leclerc T., Jennes S., Pirnay J.P., Que Y.A., Resch G., Rousseau A.F., Ravat F., Carsin H., Le Floch R. (2019). Efficacy and tolerability of a cocktail of bacteriophages to treat burn wounds infected by *Pseudomonas aeruginosa* (PhagoBurn): A randomised, controlled, double-blind phase 1/2 trial. Lancet Infect. Dis..

[55] Torres-Barceló C., Hochberg M.E. (2016). Evolutionary Rationale for Phages as Complements of Antibiotics. Trends Microbiol..

[56] Comeau A.M., Tétart F., Trojet S.N., Prère M.F., Krisch H.M. (2007). Phage-Antibiotic Synergy (PAS): β-Lactam and Quinolone Antibiotics Stimulate Virulent Phage Growth. PLoS ONE.

[57] Ryan E.M., Alkawareek M.Y., Donnelly R.F., Gilmore B.F. (2012). Synergistic phage-antibiotic combinations for the control of Escherichia coli biofilms in vitro. FEMS Immunol. Med. Microbiol..

[58] Kebriaei R., Lev K., Morrisette T., Stamper K.C., Abdul-Mutakabbir J.C., Lehman S.M., Morales S., Rybak M.J. (2020). Bacteriophage-Antibiotic Combination Strategy: An Alternative against Methicillin-Resistant Phenotypes of *Staphylococcus aureus*. Antimicrob. Agents Chemother..

[59] Morrisette T., Lev K., Kebriaei R., Abdul-Mutakabbir J.C., Stamper K.C., Morales S., Lehman S.M., Canfield G.S., Duerkop B.A., Arias C.A. (2020). Bacteriophage-Antibiotic Combinations for Enterococcus faecium with Varying Bacteriophage and Daptomycin Susceptibilities. Antimicrob. Agents Chemother..

[60] Jansen M., Wahida A., Latz S., Krüttgen A., Häfner H., Buhl E.M., Ritter K., Horz H.P. (2018). Enhanced antibacterial effect of the novel T4-like bacteriophage KARL-1 in combination with antibiotics against multi-drug resistant Acinetobacter baumannii. Sci. Rep..

[61] Cafora M., Deflorian G., Forti F., Ferrari L., Binelli G., Briani F., Ghisotti D., Pistocchi A. (2019). Phage therapy against *Pseudomonas aeruginosa* infections in a cystic fibrosis zebrafish model. Sci. Rep..

[62] Quantitative Models of Phage-Antibiotic Combination Therapy. https://www.ncbi.nlm.nih.gov/pmc/articles/PMC7002117/.

[63] Chan B.K., Turner P.E., Kim S., Mojibian H.R., Elefteriades J.A., Narayan D. (2018). Phage treatment of an aortic graft infected with *Pseudomonas aeruginosa*. Evol. Med. Public Health.

[64] Suttle C.A. (2005). Viruses in the sea. Nature.

[65] Azizian R., Nasser A., Askari H., Taheri Kalani M., Sadeghifard N., Pakzad I., Amini R., Mozaffari Nejad A.S., Azizi Jalilian F. (2015). Sewage as a rich source of phage study against *Pseudomonas aeruginosa* PAO. Biologicals.

[66] Chhibber S., Kaur P., Gondil V.S. (2018). Simple drop cast method for enumeration of bacteriophages. J. Virol. Methods..

[67] Fernández L., Gutiérrez D., García P., Rodríguez A. (2019). The Perfect Bacteriophage for Therapeutic Applications—A Quick Guide. Antibiotics.

[68] Clokie M.R., Millard A.D., Letarov A.V., Heaphy S. (2011). Phages in nature. Bacteriophage.

[69] Wright R.T., Friman V.P., Smith M.M., Brockhurst M.A. (2019). Resistance Evolution against Phage Combinations Depends on the Timing and Order of Exposure. mBio.

[70] Labrie S.J., Samson J.E., Moineau S. (2010). Bacteriophage resistance mechanisms. Nat. Rev. Microbiol..

[71] León M., Bastías R. (2015). Virulence reduction in bacteriophage resistant bacteria. Front. Microbiol..

[72] Burmeister A.R., Fortier A., Roush C., Lessing A.J., Bender R.G., Barahman R., Grant R., Chan B.K., Turner P.E. (2020). Pleiotropy complicates a trade-off between phage resistance and antibiotic resistance. Proc. Natl. Acad. Sci. USA.

[73] Chibeu A., Ceyssens P.J., Hertveldt K., Volckaert G., Cornelis P., Matthijs S., Lavigne R. (2009). The adsorption of *Pseudomonas aeruginosa* bacteriophage φKMV is dependent on expression regulation of type IV pili genes. FEMS Microbiol. Lett..

[74] Le S., Yao X., Lu S., Tan Y., Rao X., Li M., Jin X., Wang J., Zhao Y., Wu N.C. (2014). Chromosomal DNA deletion confers phage resistance to *Pseudomonas aeruginosa*. Sci. Rep..

[75] Park S.C., Shimamura I., Fukunaga M., Mori K.I., Nakai T. (2000). Isolation of Bacteriophages Specific to a Fish Pathogen, Pseudomonas plecoglossicida, as a Candidate for Disease Control. Appl. Environ. Microbiol..

[76] Forti F., Roach D.R., Cafora M., Pasini M.E., Horner D.S., Fiscarelli E.V., Rossitto M., Cariani L., Briani F., Debarbieux L. (2018). Design of a Broad-Range Bacteriophage Cocktail That Reduces *Pseudomonas aeruginosa* Biofilms and Treats Acute Infections in Two Animal Models. Antimicrob. Agents Chemother..

[77] Yang Y., Shen W., Zhong Q., Chen Q., He X., Baker J.L., Xiong K., Jin X., Wang J., Hu F. (2020). Development of a Bacteriophage Cocktail to Constrain the Emergence of Phage-Resistant *Pseudomonas aeruginosa*. Front. Microbiol..

[78] Latz S., Krüttgen A., Häfner H., Buhl E.M., Ritter K., Horz H.P. (2017). Differential Effect of Newly Isolated Phages Belonging to PB1-Like, phiKZ-Like and LUZ24-Like Viruses against Multi-Drug Resistant *Pseudomonas aeruginosa* under Varying Growth Conditions. Viruses.

[79] Chan B.K., Abedon S.T., Loc-Carrillo C. (2013). Phage cocktails and the future of phage therapy. Future Microbiol..

[80] Lehman S.M., Mearns G., Rankin D., Cole R.A., Smrekar F., Branston S.D., Morales S. (2019). Design and Preclinical Development of a Phage Product for the Treatment of Antibiotic-Resistant *Staphylococcus aureus* Infections. Viruses.

[81] Gordillo Altamirano F.L., Barr J.J. (2021). Unlocking the next generation of phage therapy: The key is in the receptors. Curr. Opin. Biotechnol..

[82] Baker P., Hill P.J., Snarr B.D., Alnabelseya N., Pestrak M.J., Lee M.J., Jennings L.K., Tam J., Melnyk R.A., Parsek M.R. (2016). Exopolysaccharide biosynthetic glycoside hydrolases can be utilized to disrupt and prevent *Pseudomonas aeruginosa* biofilms. Sci. Adv..

[83] Lin Y.W., Chang R.Y., Rao G.G., Jermain B., Han M.-L., Zhao J.X., Chen K., Wang J.P., Barr J.J., Schooley R. (2020). Pharmacokinetics/pharmacodynamics of antipseudomonal bacteriophage therapy in rats: A proof-of-concept study. Clin. Microbiol. Infect..

[84] Barbu E.M., Cady K.C., Hubby B. (2016). Phage Therapy in the Era of Synthetic Biology. Cold Spring Harb. Perspect. Biol..

[85] Smith H.W., Huggins M.B. (1982). Successful treatment of experimental Escherichia coli infections in mice using phage: Its general superiority over antibiotics. J. Gen. Microbiol..

[86] Reynaud A., Cloastre L., Bernard J., Laveran H., Ackermann H.W., Licois D., Joly B. (1992). Characteristics and diffusion in the rabbit of a phage for Escherichia coli 0103. Attempts to use this phage for therapy. Vet. Microbiol..

[87] Muir W.R., Blakemore W.S. (1960). Staphylococcal bacteriophage in experimental infection in mice. Surg. Forum.

[88] Weber-Dabrowska B., Dabrowski M., Slopek S. (1987). Studies on bacteriophage penetration in patients subjected to phage therapy. Arch. Immunol. Ther. Exp..

[89] Schultz I., Neva F.A. (1965). Relationship between Blood Clearance and Viruria after Intravenous Injection of Mice And Rats with Bacteriophage and Polioviruses. J. Immunol..

[90] Pouillot F., Chomton M., Blois H., Courroux C., Noelig J., Bidet P., Bingen E., Bonacorsi S. (2012). Efficacy of bacteriophage therapy in experimental sepsis and meningitis caused by a clone O25b:H4-ST131 Escherichia coli strain producing CTX-M-15. Antimicrob. Agents Chemother..

[91] Danis-Wlodarczyk K., Dąbrowska K., Abedon S.T. (2021). Phage Therapy: The Pharmacology of Antibacterial Viruses. Curr. Issues Mol. Biol..

[92] McVay C.S., Velásquez M., Fralick J.A. (2007). Phage Therapy of *Pseudomonas aeruginosa* Infection in a Mouse Burn Wound Model. Antimicrob. Agents Chemother..

[93] Henriksen K., Rørbo N., Rybtke M.L., Martinet M.G., Tolker-Nielsen T., Høiby N., Middelboe M., Ciofu O. (2019). P. aeruginosa flow-cell biofilms are enhanced by repeated phage treatments but can be eradicated by phage–ciprofloxacin combination: —monitoring the phage–P. aeruginosa biofilms interactions. Pathog. Dis..

[94] Bartell P.F., Geffen A., Orr T., Blakemore W.S. (1965). Staphylococcus Phage-Bacterium In Vivo Interaction. Nature.

[95] Dąbrowska K. (2019). Phage therapy: What factors shape phage pharmacokinetics and bioavailability? Systematic and critical review. Med. Res. Rev..

[96] Kantoch M. (1958). Studies on the Phagocytosis of Bacterial Viruses. I. Arch. Immunol. Ter. Dosw..

[97] Schultz I., Frohlich E. (1965). Viruria and Viraliquoria in the Dog after Intravenous Injection of T5 Bacteriophage. Proc. Soc. Exp. Biol. Med. Soc..

[98] Taylor R.P., Sutherland W.M., Martin E.N., Ferguson P.J., Reinagel M.L., Gilbert E., Lopez K., Incardona N.L., Ochs H.D. (1997). Bispecific monoclonal antibody complexes bound to primate erythrocyte complement receptor 1 facilitate virus clearance in a monkey model. J. Immunol..

[99] Inchley C.J. (1969). The actvity of mouse Kupffer cells following intravenous injection of T4 bacteriophage. Clin. Exp. Immunol..

[100] Gill J.J., Pacan J.C., Carson M.E., Leslie K.E., Griffiths M.W., Sabour P.M. (2006). Efficacy and Pharmacokinetics of Bacteriophage Therapy in Treatment of Subclinical *Staphylococcus aureus* Mastitis in Lactating Dairy Cattle. Antimicrob. Agents Chemother..

[101] Tiwari B.R., Kim S., Rahman M., Kim J. (2011). Antibacterial efficacy of lytic Pseudomonas bacteriophage in normal and neutropenic mice models. J. Microbiol..

[102] Molenaar T.M., Michon I., de Haas S.A., van Berkel T.C., Kuiper J., Biessen E.L. (2002). Uptake and processing of modified bacteriophage M13 in mice: Implications for phage display. Virology.

[103] Hájek P. (1970). The elimination of bacteriophages phiX 174 and T2 from the circulating blood of newborn precolostral pigs. Folia Microbiol. (Praha).

[104] Mukerjee S., Ghosh S.N. (1962). Localization of cholera bacterio-phage after intravenous injection. Ann. Biochem. Exp. Med..

[105] Capparelli R., Nocerino N., Iannaccone M., Ercolini D., Parlato M., Chiara M., Iannelli D. (2010). Bacteriophage therapy of Salmonella enterica: A fresh appraisal of bacteriophage therapy. J. Infect. Dis..

[106] Watanabe R., Matsumoto T., Sano G., Ishii Y., Tateda K., Sumiyama Y., Uchiyama J., Sakurai S., Matsuzaki S., Imai S. (2007). Efficacy of Bacteriophage Therapy against Gut-Derived Sepsis Caused by *Pseudomonas aeruginosa* in Mice. Antimicrob. Agents Chemother..

[107] Gabig M., Herman-Antosiewicz A., Kwiatkowska M., Los M., Thomas M.S., Wegrzyn G. (2002). The cell surface protein Ag43 facilitates phage infection of Escherichia coli in the presence of bile salts and carbohydrates. Microbiol. Read. Engl..

[108] Rus H., Cudrici C., Niculescu F. (2005). The role of the complement system in innate immunity. Immunol. Res..

[109] Dąbrowska K., Miernikiewicz P., Piotrowicz A., Hodyra K., Owczarek B., Lecion D., Kaźmierczak Z., Letarov A., Górski A. (2014). Immunogenicity studies of proteins forming the T4 phage head surface. J. Virol..

[110] Wang J., Hu B., Xu M., Yan Q., Liu S., Zhu X., Sun Z., Reed E., Ding L., Gong J. (2006). Use of bacteriophage in the treatment of experimental animal bacteremia from imipenem-resistant *Pseudomonas aeruginosa*. Int. J. Mol. Med..

[111] Altamirano F.G., Barr J.J. (2019). Phage Therapy in the Postantibiotic Era. Clin. Microbiol. Rev..

[112] Colom J., Cano-Sarabia M., Otero J., Cortés P., Maspoch D., Llagostera M. (2015). Liposome-Encapsulated Bacteriophages for Enhanced Oral Phage Therapy against Salmonella spp.. Appl. Environ. Microbiol..

[113] Malik D.J., Sokolov I.J., Vinner G.K., Mancuso F., Cinquerrui S., Vladisavljevic G.T., Clokie M.J., Garton N.J., Stapley A.F., Kirpichnikova A. (2017). Formulation, stabilisation and encapsulation of bacteriophage for phage therapy. Adv. Colloid Interface Sci..

[114] Stanford K., McAllister T.A., Niu Y.D., Stephens T.P., Mazzocco A., Waddell T.E., Johnson R.P. (2010). Oral delivery systems for encapsulated bacteriophages targeted at Escherichia coli O157:H7 in feedlot cattle. J. Food Prot..

[115] Doogue M.P., Polasek T.M. (2013). The ABCD of clinical pharmacokinetics. Ther. Adv. Drug Saf..

[116] Astudillo A., Leung S.Y., Kutter E., Morales S., Chan H.K. (2018). Nebulization effects on structural stability of bacteriophage PEV 44. Eur. J. Pharm. Biopharm..

[117] Afrin S., Gupta V. Pharmaceutical Formulation. https://www.ncbi.nlm.nih.gov/books/NBK562239/.

[118] Morrisette T., Kebriaei R., Lev K.L., Morales S., Rybak M.J. (2020). Bacteriophage Therapeutics: A Primer for Clinicians on Phage-Antibiotic Combinations. Pharmacother. J. Hum. Pharmacol. Drug Ther..

[119] Vandenheuvel D., Lavigne R., Brüssow H. (2015). Bacteriophage Therapy: Advances in Formulation Strategies and Human Clinical Trials. Annu. Rev. Virol..

[120] Kutateladze M., Adamia R. (2008). Phage therapy experience at the Eliava Institute. Méd. Mal. Infect..

[121] Rose T., Verbeken G., Vos D.D., Merabishvili M., Vaneechoutte M., Lavigne R., Jennes S., Zizi M., Pirnay J.P. (2014). Experimental phage therapy of burn wound infection: Difficult first steps. Int. J. Burns Trauma.

[122] Jennes S., Merabishvili M., Soentjens P., Pang K.W., Rose T., Keersebilck E., Soete O., François P.M., Teodorescu S., Verween G. (2017). Use of bacteriophages in the treatment of colistin-only-sensitive *Pseudomonas aeruginosa* septicaemia in a patient with acute kidney injury—A case report. Crit. Care.

[123] Marza J.S., Soothill J.S., Boydell P., Collyns T.A. (2006). Multiplication of therapeutically administered bacteriophages in *Pseudomonas aeruginosa* infected patients. Burns.

[124] Reyes A., Semenkovich N.P., Whiteson K., Rohwer F., Gordon J.I. (2012). Going viral: Next-generation sequencing applied to phage populations in the human gut. Nat. Rev. Microbiol..

[125] Reyes A., Wu M., McNulty N.P., Rohwer F.L., Gordon J.I. (2013). Gnotobiotic mouse model of phage-bacterial host dynamics in the human gut. Proc. Natl. Acad. Sci. USA.

[126] Uhr J.W., Finkelstein M.S., Baumann J.B. (1962). Antibody formation. III. The primary and secondary antibody response to bacteriophage phi X 174 in guinea pigs. J. Exp. Med..

[127] Uhr J.W., Finkelstein M.S. (1963). Antibody formation. IV. Formation of rapidly and slowly sedimenting antibodies and immunological memory to bacteriophage phi-X 174. J. Exp. Med..

[128] Hájek P., Mandel L. (1966). Antibody response of young animals to bacteriophages of different immunological behaviour: Phi X 174 and T2. Folia Microbiol..

[129] Hájek P. (1970). Neutralization of bacterial viruses by antibodies of young animals. 3. The development of the avidity of 19S and 7S neutralizing antibodies in the course of primary and secondary response in young rabbits immunized with PhiX 174 bacteriophage. Folia Microbiol..

[130] Górski A., Międzybrodzki R., Jończyk-Matysiak E., Żaczek M., Borysowski J. (2019). Phage-specific diverse effects of bacterial viruses on the immune system. Future Microbiol..

[131] Van Belleghem J.D., Clement F., Merabishvili M., Lavigne R., Vaneechoutte M. (2017). Pro- and anti-inflammatory responses of peripheral blood mononuclear cells induced by *Staphylococcus aureus* and *Pseudomonas aeruginosa* phages. Sci. Rep..

[132] Sweere J.M., Van Belleghem J.D., Ishak H., Bach M.S., Popescu M., Sunkari V., Kaber G., Manasherob R., Suh G.A., Cao X. (2019). Bacteriophage trigger antiviral immunity and prevent clearance of bacterial infection. Science.

[133] Żaczek M., Łusiak-Szelachowska M., Jończyk-Matysiak E., Weber-Dąbrowska B., Międzybrodzki R., Owczarek B., Kopciuch A., Fortuna W., Rogóż P., Górski A. (2016). Antibody Production in Response to Staphylococcal MS-1 Phage Cocktail in Patients Undergoing Phage Therapy. Front. Microbiol..

[134] Łusiak-Szelachowska M., Zaczek M., Weber-Dąbrowska B., Międzybrodzki R., Kłak M., Fortuna W., Letkiewicz S., Rogóż P., Szufnarowski K., Jończyk-Matysiak E. (2014). Phage neutralization by sera of patients receiving phage therapy. Viral Immunol..

[135] Roach D.R., Leung C.Y., Henry M., Morello E., Singh D., Di Santo J.P., Weitz J.S., Debarbieux L. (2017). Synergy between the Host Immune System and Bacteriophage Is Essential for Successful Phage Therapy against an Acute Respiratory Pathogen. Cell Host Microbe.

[136] Międzybrodzki R., Fortuna W., Weber-Dąbrowska B., Górski A. (2009). A retrospective analysis of changes in inflammatory markers in patients treated with bacterial viruses. Clin. Exp. Med..

[137] Górski A., Jończyk-Matysiak E., Łusiak-Szelachowska M., Weber-Dąbrowska B., Międzybrodzki R., Borysowski J. (2018). Therapeutic potential of phages in autoimmune liver diseases. Clin. Exp. Immunol..

[138] ClinicalTrials.gov. https://clinicaltrials.gov/ct2/show/NCT04191148.

[139] Reyes-Robles T., Dillard R.S., Cairns L.S., Silva-Valenzuela C.A., Housman M., Ali A., Wright E.R., Camilli A. (2018). Vibrio cholerae Outer Membrane Vesicles Inhibit Bacteriophage Infection. J. Bacteriol..

[140] Manning A.J., Kuehn M.J. (2011). Contribution of bacterial outer membrane vesicles to innate bacterial defense. BMC Microbiol..

[141] Issa R., Chanishvili N., Caplin J., Kakabadze E., Bakuradze N., Makalatia K., Cooper I. (2019). Antibiofilm potential of purified environmental bacteriophage preparations against early stage *Pseudomonas aeruginosa* biofilms. J. Appl. Microbiol..

[142] Gallet R., Kannoly S., Wang I.N. (2011). Effects of bacteriophage traits on plaque formation. BMC Microbiol..

[143] Parracho H.M., Burrowes B.H., Enright M.C., McConville M.L., Harper D.R. (2012). The role of regulated clinical trials in the development of bacteriophage therapeutics. J. Mol. Genet. Med. Int. J. Biomed. Res..

[144] Nilsson A.S. (2019). Pharmacological limitations of phage therapy. Ups. J. Med. Sci..

[145] Reardon S. (2014). Phage therapy gets revitalized. Nat. News.

[146] Danis-Wlodarczyk K., Vandenheuvel D., Jang H.B., Briers Y., Olszak T., Arabski M., Wasik S., Drabik M., Higgins G., Tyrrell J. (2016). A proposed integrated approach for the preclinical evaluation of phage therapy in Pseudomonas infections. Sci. Rep..

[147] Mumford R., Friman V. (2016). Bacterial competition and quorum-sensing signalling shape the eco-evolutionary outcomes of model in vitro phage therapy. Evol. Appl..

[148] Casas V., Maloy S. (2011). Role of bacteriophage-encoded exotoxins in the evolution of bacterial pathogens. Future Microbiol..

[149] Canchaya C., Fournous G., Brüssow H. (2004). The impact of prophages on bacterial chromosomes. Mol. Microbiol..

[150] Casjens S. (2003). Prophages and bacterial genomics: What have we learned so far?. Mol. Microbiol..

[151] Canchaya C., Desiere F., McShan W.M., Ferretti J.J., Parkhill J., Brüssow H. (2002). Genome analysis of an inducible prophage and prophage remnants integrated in the Streptococcus pyogenes strain SF370. Virology.

[152] Abedon S.T., Lejeune J.T. (2007). Why bacteriophage encode exotoxins and other virulence factors. Evol. Bioinform. Online.

[153] D’Herelle F., Charles C. (1930). The Bacteriophage and its Clinical Applications.

[154] Boyd E.F., Davis B.M., Hochhut B. (2001). Bacteriophage–bacteriophage interactions in the evolution of pathogenic bacteria. Trends Microbiol..

[155] Wagner P.L., Waldor M.K. (2002). Bacteriophage Control of Bacterial Virulence. Infect. Immun..

[156] Frobisher M., Brown J. (1927). Transmissible toxicogenicity of streptococci. Bull. Johns Hopkins Hosp..

[157] Freeman V.J. (1951). Studies on the Virulence of Bacteriophage-Infected Strains of Corynebacterium Diphtheriae. J. Bacteriol..

[158] Hayashi T., Matsumoto H., Ohnishi M., Terawaki Y. (1993). Molecular analysis of a cytotoxin-converting phage, φCTX, of *Pseudomonas aeruginosa*: Structure of the attP–cos–ctx region and integration into the serine tRNA gene. Mol. Microbiol..

[159] Kakabadze E., Makalatia K., Grdzelishvili N., Bakuradze N., Goderdzishvili M., Kusradze I., Phoba M.F., Lunguya O., Lood C., Lavigne R. (2018). Selection of Potential Therapeutic Bacteriophages that Lyse a CTX-M-15 Extended Spectrum β-Lactamase Producing Salmonella enterica Serovar Typhi Strain from the Democratic Republic of the Congo. Viruses.

[160] Djebara S., Maussen C., De Vos D., Merabishvili M., Damanet B., Pang K.W., De Leenheer P., Strachinaru I., Soentjens P., Pirnay J.P. (2019). Processing Phage Therapy Requests in a Brussels Military Hospital: Lessons Identified. Viruses.

[161] Vaca S., Arce J., Oliver G., Arenas D., Argüello F. (1989). FIZ15 bacteriophage increases the adhesion of pseudomonas aeruginosa to human buccal epithelial cells. Rev. Latinoam. Microbiol..

[162] Holloway B.W., Cooper G.N. (1962). Lysogenic Conversion in Pseudomonas Aeruginosa. J. Bacteriol..

[163] Matinkhoo S., Lynch K.H., Dennis J.J., Finlay W.H., Vehring R. (2011). Spray-dried respirable powders containing bacteriophages for the treatment of pulmonary infections. J. Pharm. Sci..

[164] Sahota J.S., Smith C.M., Radhakrishnan P., Winstanley C., Goderdzishvili M., Chanishvili N., Kadioglu A., O’Callaghan C., Clokie M.R. (2015). Bacteriophage Delivery by Nebulization and Efficacy Against Phenotypically Diverse *Pseudomonas aeruginosa* from Cystic Fibrosis Patients. J. Aerosol Med. Pulm. Drug Deliv..

[165] Briggiler Marcó M., Reinheimer J., Quiberoni A. (2015). Phage adsorption and lytic propagation in Lactobacillus plantarum: Could host cell starvation affect them?. BMC Microbiol..

[166] Tokman J.I., Kent D.J., Wiedmann M., Denes T. (2016). Temperature Significantly Affects the Plaquing and Adsorption Efficiencies of Listeria Phages. Front. Microbiol..

[167] Nordström K., Forsgren A., Cox P. (1974). Prevention of Bacteriophage Adsorption to *Staphylococcus aureus* by Immunoglobulin G. J. Virol..

[168] Luckey M., Neilands J.B. (1976). Iron Transport in Salmonella typhimurium LT-2: Prevention, by Ferrichrome, of Adsorption of Bacteriophages ES18 and ES18.hl to a Common Cell Envelope Receptor. J. Bacteriol..

[169] Li G., Shen M., Yang Y., Le S., Li M., Wang J., Zhao Y., Tan Y., Hu F., Lu S. (2018). Adaptation of *Pseudomonas aeruginosa* to Phage PaP1 Predation via O-Antigen Polymerase Mutation. Front. Microbiol..

[170] Hall-Stoodley L., Costerton J.W., Stoodley P. (2004). Bacterial biofilms: From the natural environment to infectious diseases. Nat. Rev. Microbiol..

[171] Chaudhry W.N., Concepción-Acevedo J., Park T., Andleeb S., Bull J.J., Levin B.R. (2017). Synergy and Order Effects of Antibiotics and Phages in Killing *Pseudomonas aeruginosa* Biofilms. PLoS ONE.

[172] Sánchez-Romero M.A., Casadesús J. (2014). Contribution of phenotypic heterogeneity to adaptive antibiotic resistance. Proc. Natl. Acad. Sci. USA.

[173] Akanda Z.Z., Taha M., Abdelbary H. (2018). Current review—The rise of bacteriophage as a unique therapeutic platform in treating peri-prosthetic joint infections. J. Orthop. Res..

[174] Clinical Microbiology Reviews: Prosthetic Joint Infection. https://cmr.asm.org/content/27/2/302.

[175] Ferry T., Boucher F., Fevre C., Perpoint T., Chateau J., Petitjean C., Josse J., Chidiac C., L’hostis G., Leboucher G. (2018). Innovations for the treatment of a complex bone and joint infection due to XDR *Pseudomonas aeruginosa* including local application of a selected cocktail of bacteriophages. J. Antimicrob. Chemother..

[176] Burgener E.B., Sweere J.M., Bach M.S., Secor P.R., Haddock N., Jennings L.K., Marvig R.L., Johansen H.K., Rossi E., Cao X. (2019). Filamentous bacteriophages are associated with chronic Pseudomonas lung infections and antibiotic resistance in cystic fibrosis. Sci. Transl. Med..

[177] Rosenfeld M., Gibson R.L., McNamara S., Emerson J., Burns J.L., Castile R., Hiatt P., McCoy K., Wilson C.B., Inglis A. (2001). Early pulmonary infection, inflammation, and clinical outcomes in infants with cystic fibrosis. Pediatr. Pulmonol..

[178] Emerson J., Rosenfeld M., McNamara S., Ramsey B., Gibson R.L. (2002). Pseudomonas aeruginosa and other predictors of mortality and morbidity in young children with cystic fibrosis. Pediatr. Pulmonol..

[179] Mitri C., Xu Z., Bardin P., Corvol H., Touqui L., Tabary O. (2020). Novel Anti-Inflammatory Approaches for Cystic Fibrosis Lung Disease: Identification of Molecular Targets and Design of Innovative Therapies. Front. Pharmacol..

[180] ClinicalTrials.gov. https://clinicaltrials.gov/ct2/show/NCT04596319.

[181] Lin D.M., Koskella B., Lin H.C. (2017). Phage therapy: An alternative to antibiotics in the age of multi-drug resistance. World J. Gastrointest. Pharmacol. Ther..

